# Complete mitochondrial genome of the clearwing moth *Synanthedon bicingulata* (Lepidoptera: Sesiidae)

**DOI:** 10.1080/23802359.2024.2427095

**Published:** 2024-11-12

**Authors:** Woo Jin Kim, Seung Hyun Lee, Jeong Sun Park, Iksoo Kim

**Affiliations:** a Department of Forest-Bio, Jeollanam-do Forest Research Institute, Naju-si, Republic of Korea; b Department of Applied Biology, College of Agriculture & Life Sciences, Chonnam National University, Gwangju, Republic of Korea

**Keywords:** *Synanthedon bicingulata*, clearwing moth, mitochondrial genome, gene arrangement, intergenic spacer sequences, phylogeny

## Abstract

The clearwing moth, *Synanthedon bicingulata* Staudinger, 1887 (Lepidoptera: Sesiidae), is a serious pest that infests cherry trees. This species, which is distributed in South Korea, was once regarded as *S. hector* found in Japan, but renamed as *S. bicingulata* based on morphology. Molecular data for this species are not available. Hence, we sequenced the 16,255-bp long complete mitochondrial genome (mitogenome) of *S. bicingulata*. Phylogenetic analyses in the Cossoidea superfamily supported monophyly of the *Synanthedon* genus and Synanthedonini tribe. This newly sequenced mitogenome of *S. bicingulata* will be useful for studies in a diverse field of evolutionary study.

## Introduction

*Synanthedon bicingulata* Staudinger, 1887 (Lepidoptera: Sesiidae) is a major pest, which in its larval stage, causes wood-boring damage to trees of the *Prunus* genus, such as plum, peach, and cherry. *S. bicingulata* is a major cause for concern in South Korea because cherry trees account for the highest proportion of planted tree species in the natural landscape (Korea Forest Service 2023).

*S. bicingulata*, which is distributed in South Korea, China, and Russia, has been confused with its congener *S. hector* Butler, 1878 that is distributed in Japan because of the superficial similarities between them (Matsumura 1931). This was later revised, and the species was renamed *S. bicingulata* based on the morphological characteristics of the adults, including genital features, as well as those of the larvae and pupae (Arita et al. 2004; Lee et al. 2004). However, no sequencing information for *S. bicingulata* is available. Moreover, a very limited number of *cox1* sequences for the tribe Synanthedonini, in which *S. bicingulata* is included, and the mitochondrial genomes (mitogenomes) of only four of approximately 287 *Synanthedon* species (Pühringer and Kallies 2004; Zheng et al. 2022) are available as public data to date.

In this study, we sequenced the complete mitogenome of *S. bicingulata* and scrutinized the major features of the genome to illustrate the evolutionary characteristics of the species. We also compared the baseline genomic characteristics and performed phylogenetic analysis in the context of the Cossoidea superfamily, to which *S. bicingulata* belongs. The information regarding the newly sequenced complete mitogenome of *S. bicingulata* will be useful for species identification and phylogenetic study of the *Synanthedon* genus and Synanthedonini tribe, which include a substantial number of species.

## Materials and methods

**Figure 1.**
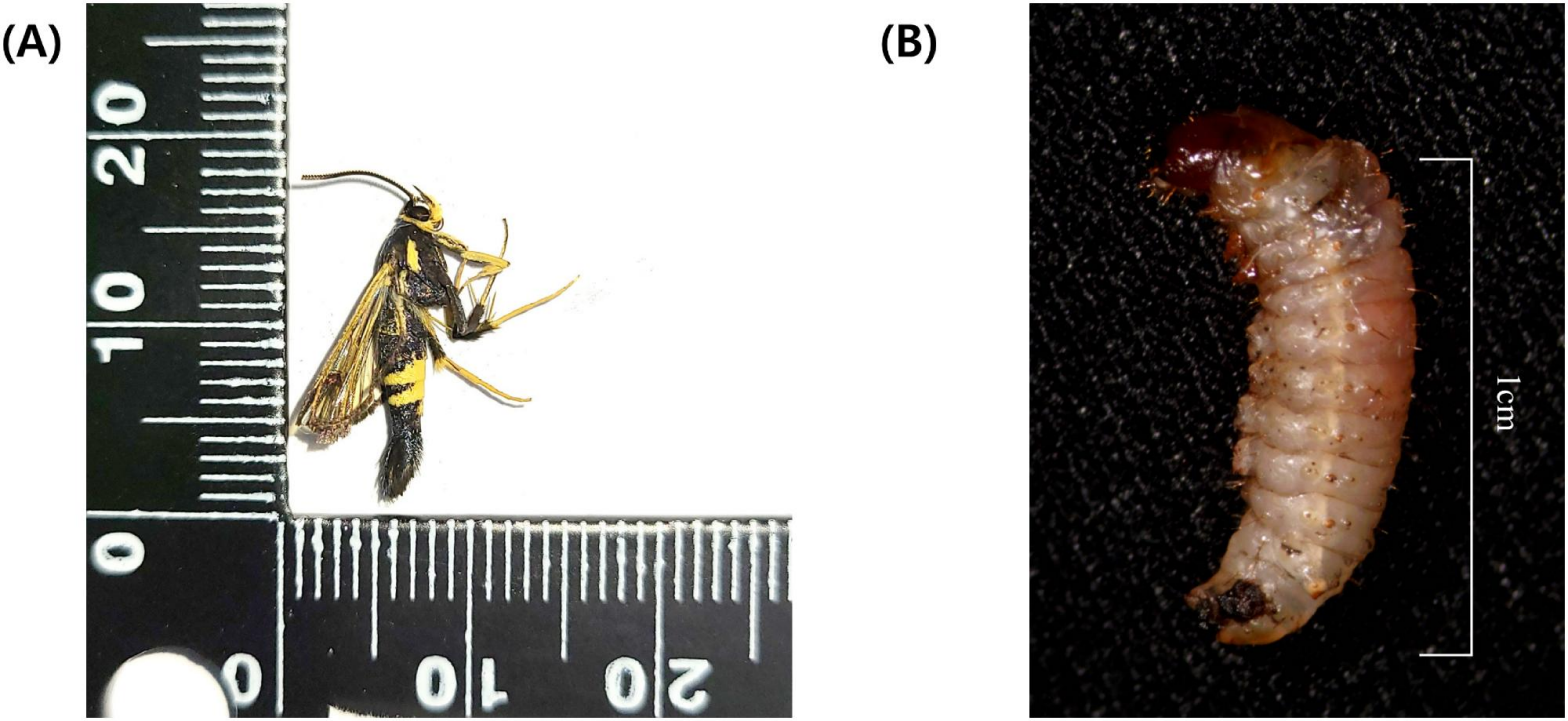
Images of *Synanthedon bicingulata* Staudinger, 1887. (A) Male adult. (B) Larva caught from underneath the bark of a cherry tree. The photos were taken by the author, Woo Jin Kim.

In April 2023, male adult clearwing moths (*S. bicingulata*) were collected from Suncheon-si, South Korea (34°58′20.9″ N, 127°23′34.4″ E) using a species-specific sex pheromone trap (GreenAgrotech, Gyeongsan, South Korea). Several larvae feeding underneath the bark of a cherry tree that was leaking dark brown gum were also collected (Figure 1). The adults were identified by one of the authors (Iksoo Kim) based on the morphological characteristics, including the vivid yellow stripes that are found distally on the fourth and fifth abdominal tergites (Lee et al. 2004).

Total DNA was extracted from the hind leg of the male adult insects using the Wizard™ Genomic DNA Purification Kit (Promega, Madison, WI). This voucher specimen and leftover DNA were deposited at the Jeollanam-do Forest Research Institute, Jeollanam-do, South Korea under the accession number SC(II)-0426 (Woo Jin Kim, littleboots@korea.kr).

The complete mitogenome was amplified into three long overlapping fragments (LFs). Subsequently, 26 short overlapping fragments were amplified using the LFs as templates (Figure S1). The primers reported by Kim et al. (2012) and two newly designed primers were utilized (Table S1). Sequencing was conducted using Sanger’s sequencing method. The full-length mitogenome was assembled using the SeqMan software from the DNASTAR package (SeqMan NGen^®^, version 13.0, DNASTAR, Madison, WI) by aligning overlapping sequences of adjacent fragments. Individual genes and the A + T-rich region were annotated by aligning homologous sequences of known full-length lepidopteran mitogenomes using MAFFT, version 7 (Katoh and Standley 2013). The nucleotide sequences of protein-coding genes (PCGs) were translated based on the invertebrate mitochondrial DNA genetic code to check for any unconventional sequences, possibly from nuclear-embedded mitochondrial DNA sequences. tRNA genes were identified using tRNAscan-SE, version 2.0 (Lowe and Eddy 1997). The sequence data were deposited in the GenBank database under the accession number PP622747.

A total of 12 mitogenomes in the Cossoidea superfamily were downloaded from GenBank, and the baseline information of *S. bicingulata* and the Cossoidea members was analyzed. Phylogenetic analysis was conducted using the nucleotide sequences of 13 PCGs and two rRNAs of the Cossoidea members (13,592 bp including gaps) with the inclusion of an outgroup, *Leguminivora glycinivorella*, belonging to the Tortricoidea superfamily. For this, Bayesian inference (Ronquist et al. 2012) and IQ-TREE (Nguyen et al. 2015), integrated into PhyloSuite, version 1.2.3 (Xiang et al. 2023) were used. An optimal partitioning scheme (nine partitions) and substitution model were determined using PartitionFinder 2 with the Greedy algorithm (Lanfear et al. 2017).

## Results

**Figure 2.**
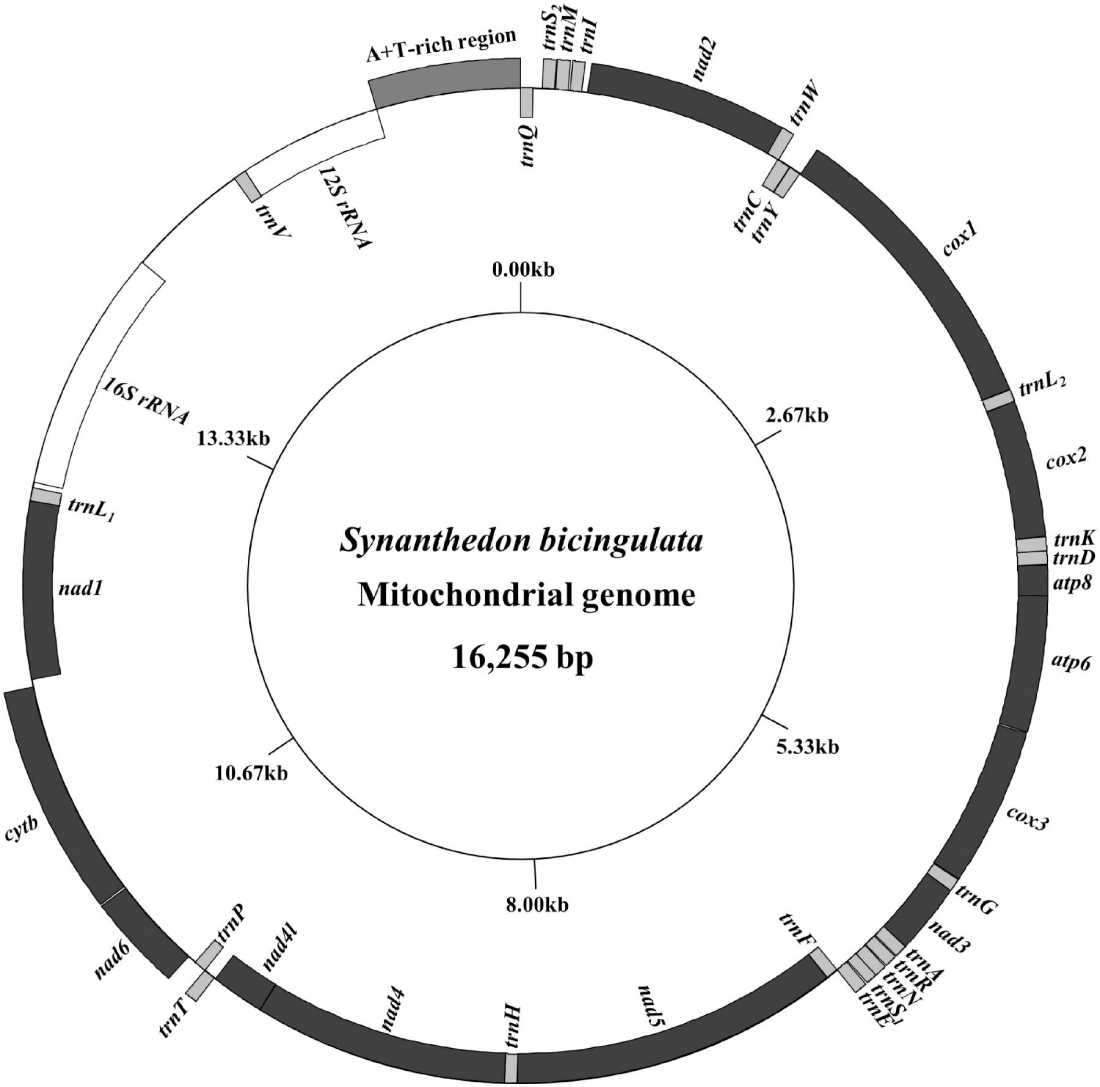
Circular map of the mitochondrial genome of *Synanthedon bicingulata* obtained using the GenomeVx tool (http://wolfe.ucd.ie/GenomeVx/). tRNA abbreviations follow the IUPAC-IUB one-letter code. *trnL_1_*, *trnL_2_*, *trnS_1_*, and *trnS_2_*, denote *tRNA^Leu^*(CUN), *tRNA^Leu^*(UUR), *tRNA^Ser^*(AGN), and *tRNA^Ser^*(UCN), respectively. Gene names outside the circular map indicate transcription in a clockwise direction, excluding the A + T-rich region, while those inside the map indicate transcription in a counter-clockwise direction.

The 16,255-bp long complete mitogenome of *S. bicingulata* contains two rRNAs, 22 tRNAs, 13 PCGs, and an A + T-rich region (Figure 2). *cox1* starts with the CGA codon and *atp8*, with the TTG codon. The remaining PCGs start with typical ATN codons. *cox1*, *nad5*, and *nad4* have the incomplete stop codon T, whereas the remaining PCGs terminate with TAA (Table 1). The *S. bicingulata* mitogenome has a rearranged gene block (*trnQ*-*trnS_2_*-*trnM*-*trnI* [underlined gene names indicate a counter-clockwise transcriptional direction]) between the A + T-rich region and *nad2*, differing from the typical arrangement of *trnM*-*trnI*-*trnQ* found in a majority of the lepidopterans (Figure S2). The A/T content was highest in the A + T-rich region (94.1%), followed by that in *16S rRNA* (85.1%), *12S rRNA* (85.0%), 22 tRNAs (82.4%), whole genome (79.7%), and 13 PCGs (76.7%) (Table S2).

**Table 1.** Summary of *Synanthedon bicingulata* mitochondrial genome.

Gene	Nucleotide number	Size (bp)	Anticodon	Codon		O/S
				Start	Stop	
*trnQ*	1–67	67	TTG	–	–	
*trnS_2_*	114–178	65	TGA	–	–	−46
*trnM*	181–247	67	CAT	–	–	−2
*trnI*	257–320	64	GAT	–	–	−9
*nad2*	351–1343	993	–	ATA	TAA	−30
*trnW*	1345–1411	67	TCA	–	–	−1
*trnC*	1404–1478	75	GCA	–	–	+8
*trnY*	1479–1542	64	GTA	–	–	
*cox1*	1546–3076	1531	–	CGA	T–tRNA	−3
*trnL_2_*	3077–3140	64	TAA	–	–	
*cox2*	3141–3821	681	–	ATA	TAA	
*trnK*	3823–3893	71	CTT	–	–	−1
*trnD*	3894–3960	67	GTC	–	–	
*atp8*	3961–4119	159	–	TTG	TAA	
*atp6*	4113–4790	678	–	ATG	TAA	+7
*cox3*	4796–5590	795	–	ATG	TAA	−5
*trnG*	5595–5658	64	TCC	–	–	−4
*nad3*	5659–6012	354	–	ATT	TAA	
*trnA*	6013–6076	64	TGC	–	–	
*trnR*	6082–6144	63	TCG	–	–	−5
*trnN*	6151–6215	65	GTT	–	–	−6
*trnS_1_*	6214–6272	59	GCT	–	–	+2
*trnE*	6279–6343	65	TTC	–	–	−6
*trnF*	6344–6410	67	GAA	–	–	
*nad5*	6411–8142	1732	–	ATT	T–tRNA	
*trnH*	8143–8209	67	GTG	–	–	
*nad4*	8210–9551	1342	–	ATG	T–tRNA	
*nad4l*	9552–9836	285	–	ATG	TAA	
*trnT*	9842–9906	65	TGT	–	–	−5
*trnP*	9906–9971	66	TGG	–	–	+1
*nad6*	10,018–10,503	486	–	ATA	TAA	−46
*cytb*	10,511–11,662	1152	–	ATG	TAA	−7
*nad1*	11,700–12,635	936	–	ATG	TAA	−37
*trnL_1_*	12,637–12,704	68	TAG	–	–	−1
*16S rRNA*	12,735–14,022	1288	–	–	–	−30
*trnV*	14,671–14,737	67	TAC	–	–	−648
*12S rRNA*	14,738–15,496	759	–	–	–	
A + T-rich region	15,497–16,255	759	–	–	–	

Gene names that are not underlined indicate a clockwise transcriptional direction, whereas those that are underlined indicate a counter-clockwise transcriptional direction. tRNAs abbreviations follow the IUPAC-IUB one-letter code. *trnL_1_*, *trnL_2_*, *trnS_1_*, and *trnS_2_* denote *tRNA^Leu^*(CUN), *tRNA^Leu^*(UUR), *tRNA^Ser^*(AGN), and *tRNA^Ser^*(UCN), respectively. O/S, number of overlapping (+)/intergenic spacer (−) sequences.

The *S. bicingulata* mitogenome contains unusually long intergenic spacer sequences at the *16S rRNA* and *trnV* junction (648 bp; Table 1), comprising seven tandemly repeated sequences that are near-even at 60–64 bp, encompassed at each end by non-repeat sequences (Figure S3).

**Figure 3.**
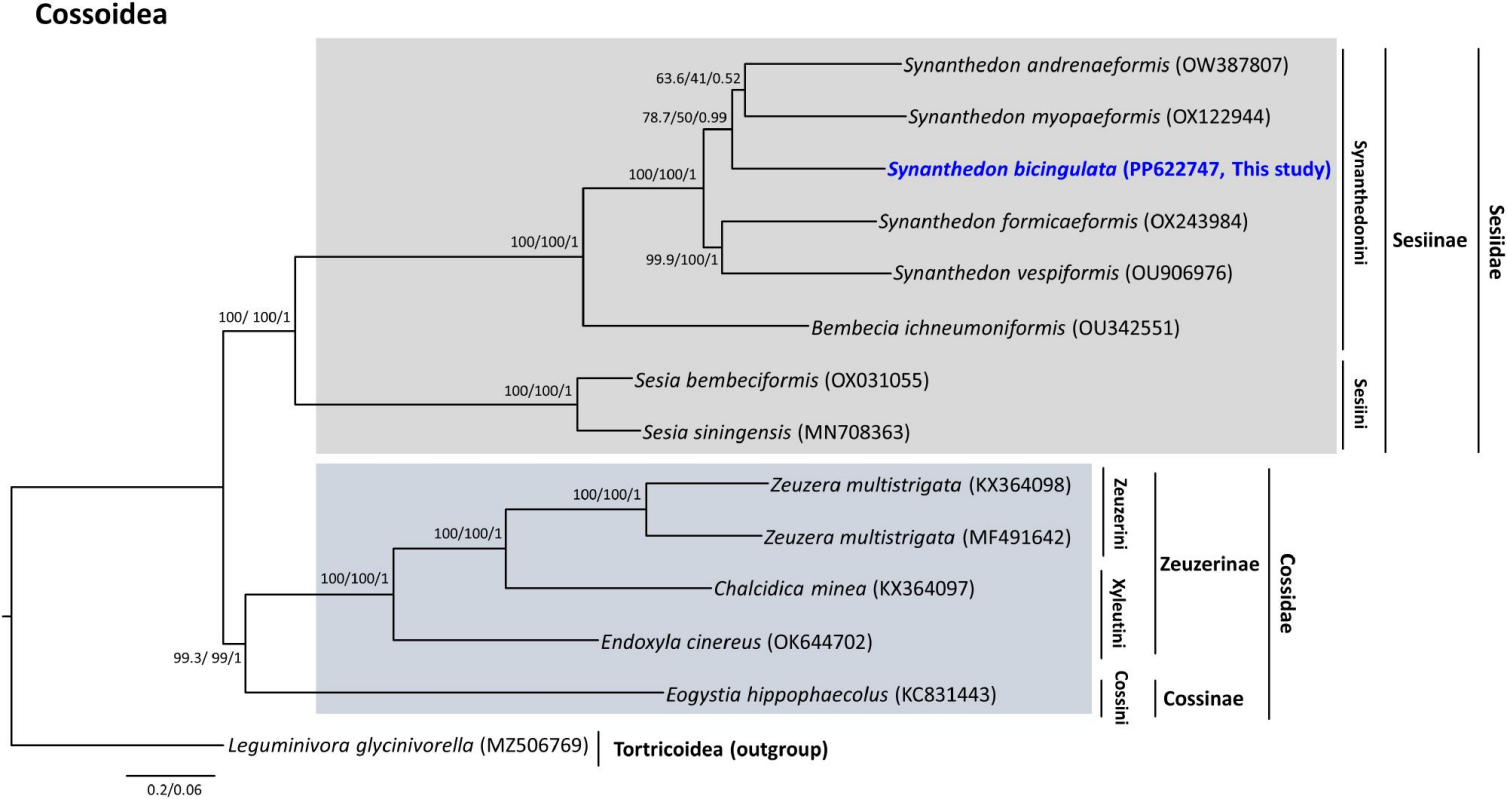
Phylogeny of 13 mitochondrial genomes of members of the Sesiidae and Cossidae families in the Cossoidea superfamily, including *Synanthedon bicingulata*, derived using maximum-likelihood and Bayesian’s inference methods. The numbers at each node are Shimodaira–Hasegawa-like approximate likelihood ratio test (SH-aLRT)/Ultrafast bootstrap (UFBoot)/Bayesian’s posterior probabilities (BPPs). The scale bar indicates the number of substitutions per site. *Leguminivora glycinivorella* (MZ506769; unpublished) belonging to the Tortricoidea superfamily, was used as an outgroup. The following sequences were used: *Synanthedon bicingulata* (PP622747; this study), *Synanthedon vespiformis* (OU906976; Boyes and Lees 2022), *Synanthedon formicaeformis* (OX243984; Langdon and Fagan 2023), *Synanthedon andrenaeformis* (OW387807; unpublished), *Synanthedon myopaeformis* (OX122944; unpublished), *Bembecia ichneumoniformis* (OU342551; Boyes 2023), *Sesia bembeciformis* (OX031055; Boyes and Langdon 2023), *Sesia siningensis* (MN708363; Yan et al. 2019), *Eogystia hippophaecolus* (KC831443; Gong et al. 2014), *Chalcidica minea* (KX364097; Li et al. 2018), *Endoxyla cinereus* (OK644702; unpublished), *Zeuzera multistrigata* (KX364098; Li et al. 2018), *Zeuzera multistrigata* (MF491642; Kim et al. 2017), and *Leguminivora glycinivorella* (MZ506769; unpublished).

Phylogenetic analysis showed that the *Synanthedon* genus, Synanthedonini tribe, Sesiinae subfamily, and Sesiidae family, to which *S. bicingulata* belongs, are monophyletic groups with the highest nodal supports (Figure 3). *S. bicingulata* was placed as the sister to the group containing *S. anderenaeformis* and *S. myopaeformis*, but nodal support for this relationship was extremely low. Currently, the *Synanthedon* genus comprises 287 species (Pühringer and Kallies 2004; Zheng et al. 2022). Thus, the limited number of species included in this study may be the reason for this low support. Further studies scrutinizing an extended taxon diversity are required for better understanding.

## Discussion and conclusions

The length and A + T content of the whole genome, genes, and the A + T-rich region are well within the range found in other members of the Cossoidea superfamily (Table S2). The A + T content of a majority of the Cossoidea members is higher in *12S rRNA* than that in *16S rRNA*, but *Zeuzera* species in the Zeuzerinae subfamily and the current *S. bicingulata* have higher A + T content in *16S rRNA* (Table S2).

The rearranged gene block found in *S. bicingulata* mitogenome (*trnQ*-*trnS_2_*-*trnM*-*trnI*) differs from the typical *trnM*-*trnI*-*trnQ* arrangement at the same junction, which is regarded as a derived character in lepidopterans, with a few exceptions (Figure S2; Cao et al. 2012; Timmermans et al. 2014). The translocation of *trnQ* and *trnS_2_* could be the prime events involved in this new arrangement. Considering all six species, including *S. bicingulata* in the Synanthedonini tribe, have the same rearrangement at this junction, it could be a shared-derived character for this tribe. However, further studies are required to confirm this hypothesis.

The 648-bp long intergenic spacer sequences containing seven near-even tandem repeat sequences between *16S rRNA* and *trnV* are unique (Figure S3) because other species in the Cossoidea superfamily have a maximum of 38-bp long intergenic spacer sequences at the same junction without any repeat sequence (*Endoxyla cinereus*; GenBank accession number OK644702; unpublished). This region could potentially be a prime target site for species identification. Previous phylogenetic studies involving *Synanthedon* and Synanthedonini did not include *S. bicingulata* (Liang et al. 2022; Cognato et al. 2023). Thus, the current *S. bicingulata* mitogenome will be useful for future phylogenetic analysis of *Synanthedon* and Synanthedonini. Moreover, the *S. bicingulata* mitogenome sequences, particularly the 648-bp long intergenic spacer sequences, will be useful for species identification.

## Supplementary Material

Table S1_List of primers.docx

Figure S3_Repeat sequences.pdf

Table S2_Characteristics of Cossoidea.docx

Figure S1_PCR.pdf

Figure S2_Linear arrangement_Revised.pdf

## Data Availability

The genome sequence data that support the findings of this study are openly available in GenBank of NCBI (https://www.ncbi.nlm.nih.gov) under the accession number PP622747.
