# Supplementary material for: Complete mitochondrial genome of the clearwing moth Synanthedon bicingulata (Lepidoptera: Sesiidae)
Source: Mitochondrial DNA B Resour. 2024 Nov 12;9(11):1528–32. doi: 10.1080/23802359.2024.2427095 (PMC11562021; doi:10.1080/23802359.2024.2427095)
Supplement: Table S1_List of primers.docx [file TMDN_A_2427095_SM2355.docx]

**Table S1.** List of primers used to amplify and sequence the mitochondrial genome of *Synanthedon bicingulata*

| Fragment name | Primer name^a^ | Direction^b^ | Sequence (5’-3’) | Nucleotide position^c^ |
| --- | --- | --- | --- | --- |
| *Long fragment* |  |  |  |  |
| LF1 | Lep-COI-F2 | F | ATAGTTATACCTATTATAATTGG | 1741-1763 |
| LF1 | Lep-ND4-R2 | R | GCTCATGTTGAAGCTCCTGT | 8911-8930 |
| LF2 | Lep-ND5-F2 | F | CGAATATCTTGAATATCATTTATTA | 6367-6391 |
| LF2 | Lep-lrRNA-R2 | R | GTATCTTGTGTATCAGAGTTTA | 13851-13872 |
| LF3 | Lep-lrRNA-F1 | F | TGTAAGATTTTAATGATCGAACAGAT | 12852-12877 |
| LF3 | Lep-COI-R1 | R | CTTCAGGATGACCAAAAAATC | 2243-2263 |
| *Short fragment* |  |  |  |  |
| SF1 | LF03-S05-F2 | F | TWAAAGGATTATTCTGATAG | 271-290 |
| SF1 | LF03-S05-R2 | R | CAWCCTAAATTATTAATWGAWGA | 858-880 |
| SF2 | LF03-S06-F2 | F | ATTRTWGAAGGWTTATCWTG | 675-694 |
| SF2 | LF03-S06-R1 | R | GATATAAAATTGCAAATTTTAAG | 1431-1453 |
| SF3 | COIF | F | CTTAAAATTTGCAATTTTATATC | 1431-1453 |
| SF3 | Lep-COI-R1 | R | CTTCAGGATGACCAAAAAATC | 2243-2263 |
| SF4 | LF01-S01-F2 | F | TTACAACAATTATTAATATACG | 2030-2051 |
| SF4 | LF01-S01-R2 | R | GTCGAGGTATTCCTGCTA | 2834-2851 |
| SF5 | LF01-S02-F2 | F | ACWGTAGGAGGATTAACAGG | 2581-2600 |
| SF5 | LF01-S02-R2 | R | GTTCAAATTAATTCAATTATTTG | 3315-3337 |
| SF6 | LF01-S03-F2 | F | TAGAAATGGCAACWTGATC | 3136-3154 |
| SF6 | LF01-S03-R1 | R | CTTGCTTTCAGTCATCTAAT | 3824-3843 |
| SF7 | LF01-S04-F1 | F | CAGGTCGWTTAAATCAAAC | 3667-3685 |
| SF7 | LF01-S04-R2 | R | GTTCCTTGDGGAATTATATG | 4497-4516 |
| SF8 | LF01-S05-F1 | F | TTATTTTCAATTTTTGATCC | 4125-4144 |
| SF8 | LF01-S05-R2 | R | CCAATTTCAATATTAGGDGATA | 5121-5142 |
| SF9 | LF01-S06-F2 | F | GTWGATTATAGHCCWTGACC | 4835-4854 |
| SF9 | LF01-S06-R2 | R | GATTGGAAGTCAAATATACT | 5612-5631 |
| SF10 | Synanthedon-SF10-F1 | F | ATTTCTTTTAATTTGCTTAATTCG | 5443-5466 |
| SF10 | LF01-S07-R1 | R | CAATTTTATCATTAACAGTGA | 6308-6328 |
| SF11 | LF01-S08-F2 | F | GAAATCAAAATATATTAAATTG | 5981-6002 |
| SF11 | LF01-S08-R1 | R | TGATTTATACCTARWTTATCWAC | 6626-6648 |
| SF12 | LF01-S09-F1 | F | AWAHTTCTCTTCAACCYAWATC | 6549-6570 |
| SF12 | LF01-S09-R2 | R | GCTTTATCWACTTTAAGWCA | 7304-7323 |
| SF13 | LF01-S10-F1 | F | TCYTTWGAATAAAAYCCAG | 7052-7070 |
| SF13 | LF01-S10-R1 | R | GATGGDTTAGGDTTAGTTTCTT | 7773-7794 |
| SF14 | LF01-S11-F1 | F | AAAAAATATAATTTCAWCTHCC | 7632-7653 |
| SF14 | LF01-S11-R2 | R | GAGCTGGDTATAGATTATAT | 8358-8377 |
| SF15 | LF01-S12-F1 | F | ATATTTTTGAYHCCACAAATC | 8164-8184 |
| SF15 | LF01-S12-R1 | R | CAGGTTCAATAATTTTAGC | 8890-8908 |
| SF16 | LF02-S01-F1 | F | TTATAATACCHCCAATWAC | 8678-8696 |
| SF16 | LF02-S01-R1 | R | GGTTTAATTTTATTAAGAATTTG | 9352-9374 |
| SF17 | LF02-S02-F1 | F | ATATTAAAGTAGGAATTAAWC | 9178-9198 |
| SF17 | LF02-S02-R2 | R | TAATTTTGGAGATTATWGAT | 9928-9947 |
| SF18 | LF02-S03-F1 | F | CCTAAAGCHCCYTCACAAAC | 9623-9642 |
| SF18 | LF02-S03-R2 | R | GGTAAATCAATTAAWGATCYAT | 10566-10587 |
| SF19 | LF02-S04-F2 | F | TNTCAAGAATTGCHTCWAATG | 10178-10198 |
| SF19 | LF02-S04-R1 | R | GATATTTGTCCYCAAGGTA | 10920-10938 |
| SF20 | LF02-S05-F2 | F | TATHTHCATATTGGACGAGG | 10805-10824 |
| SF20 | LF02-S05-R2 | R | CCAATTCAWGTTAATAAAAT | 11519-11538 |
| SF21 | LF02-S06-F2 | F | ACHCCHRTTCATATTCAACC | 11312-11331 |
| SF21 | LF02-S06-R2 | R | GCTGAAACTAATCGAACTC | 12026-12044 |
| SF22 | LF02-S07-F2 | F | AAAGCAAATCCCCCTCTTC | 11950-11968 |
| SF22 | LF02-S07-R2 | R | CTGAGTTCAAACCGGTGTRA | 12828-12847 |
| SF23 | LF02-S08-F1 | F | TCTAATAAAGTTAAAAAAGC | 12544-12563 |
| SF23 | LF02-S08-R2 | R | CACTTGTTTATCAAAAACATGTC | 13360-13382 |
| SF24 | LF03-S02-F1 | F | ATTATGCTACCTTTGTACAGTC | 13276-13297 |
| SF24 | LF03-S02-R1 | R | GTATTTCATTTACATTGAAAAGA | 14693-14715 |
| SF25 | LF03-S03-F3 | F | CTCTGATACACAAGATAC | 13855-13872 |
| SF25 | LF03-S03-R3 | R | CCAGCAGTTGCGGTTAAAC | 15317-15335 |
| SF26 | Synanthedon-SF26-F1 | F | TCTAAATGAACTAAAATACCG | 15084-15104 |
| SF26 | LF03-S04-R1 | R | CTATCAGAATAATCCTTTWA | 271-290 |

^a^Most primers were adapted from Kim et al. (2012), but Synanthedon-SF10-F1 and Synanthedon-SF26-F1 were newly designed in this study using available *Synanthedon* mitochondrial genome sequences.

^b^F and R, forward and reverse direction of transcription

^c^Nucleotide positions are with respect to *Synanthedon bicingulata* mitochondrial genome.
