# Supplementary material for: Complete mitochondrial genome of the clearwing moth Synanthedon bicingulata (Lepidoptera: Sesiidae)
Source: Mitochondrial DNA B Resour. 2024 Nov 12;9(11):1528–32. doi: 10.1080/23802359.2024.2427095 (PMC11562021; doi:10.1080/23802359.2024.2427095)
Supplement: Figure S3_Repeat sequences.pdf [file TMDN_A_2427095_SM2354.pdf]

(A)

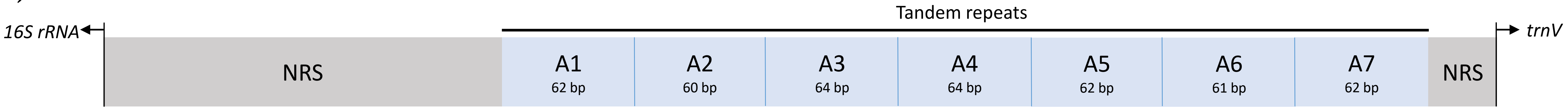


(B)

```
A1  AAAAAATTTTTT-C-TTTTTTTTT-AATTTT-CCCC-TTTTTTT--AATTTTTTTTGACTTTTTCATTC
A2  AAAAAATTTTTT-CCTTTTCTTT--AATTTT-CC---TTTTTTTT-AATTTTTTT-GACTTTTTCATTC
A3  AAAAAATTTTTTTC-TTTTTTTTTTAATTTT-CCC--TTTTTTTTTAATTTTTTT-GACTTTTTCACCT
A4  AAAAAATTTTTTTC-TTACTTTTT-GATTTT-CCCC-TTTTTTTT-AATTTTTTTTGACTTTTTCACCT
A5  AAAAAATTTTTT-C-TTTTTTTT--AATTTTTCCCC-TTTTTTTT-AATTTTCTT-GACTTTTTCATTC
A6  AAAAAATTTTTT-C-TTTTTTTTT-AATTTT-CCCCCTTTTTT---AATTTTTTT-GACTTTTTCATTC
A7  AAAAA-GTTTTTTC-TTTTTTCTTTGATTTT-CCCC-TTTTTT---AATTTTTTTTGACTTTTTCACCT
    *****  ***** * **   * *   ***** **   ******   ****** ** **********
```

**Figure S3.** Schematic map of repeat sequences of *Synanthedon bicingulata* found in intergenic spacer sequences between *16S rRNA* and *trnV.* (A) Arrangement of repeat sequences. (B) Alignment of repeat sequences. NRS, non-repeat sequence; *, consensus sequence.
