# Supplementary material for: Complete mitochondrial genome of the clearwing moth Synanthedon bicingulata (Lepidoptera: Sesiidae)
Source: Mitochondrial DNA B Resour. 2024 Nov 12;9(11):1528–32. doi: 10.1080/23802359.2024.2427095 (PMC11562021; doi:10.1080/23802359.2024.2427095)
Supplement: Table S2_Characteristics of Cossoidea.docx [file TMDN_A_2427095_SM2353.docx]

Table S2. Characteristics of Cossoidea mitochondrial genomes

| Taxon | Size (bp) | A/T (%) | PCG^a^ | | *12S rRNA* | | *16S rRNA* | | tRNA | | A+T-rich region | | GenBank accession number | Reference |
| --- | --- | --- | --- | --- | --- | --- | --- | --- | --- | --- | --- | --- | --- | --- |
|  |  |  | Number of codons^a^ | A/T (%) | Size (bp) | A/T (%) | Size (bp) | A/T (%) | Size (bp) | A/T (%) | Size (bp) | A/T (%) |  |  |
| Cossoidea |  |  |  |  |  |  |  |  |  |  |  |  |  |  |
| Cossidae |  |  |  |  |  |  |  |  |  |  |  |  |  |  |
| Zeuzerinae |  |  |  |  |  |  |  |  |  |  |  |  |  |  |
| Zeuzerini |  |  |  |  |  |  |  |  |  |  |  |  |  |  |
| *Zeuzera multistrigata* | 15,260 | 78.9 | 3,716 | 76.9 | 759 | 84.7 | 1,346 | 84.6 | 1,465 | 80.4 | 374 | 93.9 | MF491642 | Kim et al. (2017) |
| *Zeuzera multistrigata* | 15,320 | 78.9 | 3,722 | 76.9 | 786 | 83.5 | 1,346 | 83.6 | 1,476 | 81.1 | 356 | 93.3 | KX364098 | Li et al. (2018) |
| Xyleutini |  |  |  |  |  |  |  |  |  |  |  |  |  |  |
| *Endoxyla cinereus* | 15,285 | 81.6 | 3,717 | 80.3 | 766 | 85.8 | 1,298 | 84.7 | 1,474 | 81.8 | 308 | 95.1 | OK644702 | Unpublished |
| *Chalcidica minea* | 15,490 | 82.1 | 3,718 | 80.5 | 780 | 86.0 | 1,379 | 84.8 | 1,487 | 82.7 | 354 | 95.8 | KX364097 | Li et al. (2018) |
| Cossinae |  |  |  |  |  |  |  |  |  |  |  |  |  |  |
| Cossini |  |  |  |  |  |  |  |  |  |  |  |  |  |  |
| *Eogystia hippophaecolus* | 15,431 | 78.4 | 3,731 | 76.2 | 779 | 85.5 | 1,364 | 82.4 | 1,488 | 81.1 | 317 | 92.7 | KC831443 | Gong et al. (2014) |
| Sesiidae |  |  |  |  |  |  |  |  |  |  |  |  |  |  |
| Sesiinae |  |  |  |  |  |  |  |  |  |  |  |  |  |  |
| Sesiini |  |  |  |  |  |  |  |  |  |  |  |  |  |  |
| *Sesia siningensis* | 15,454 | 79.7 | 3,692 | 77.4 | 773 | 86.0 | 1,322 | 83.4 | 1,460 | 81.1 | 378 | 98.2 | MN708363 | Yan et al. (2020) |
| *Sesia bembeciformis* | 16,056 | 80.2 | 3,692 | 77.2 | 774 | 85.5 | 1,320 | 83.6 | 1,466 | 81.2 | 1,005 | 97.6 | OX031055 | Boyes and Langdon (2023) |
| Synanthedonini |  |  |  |  |  |  |  |  |  |  |  |  |  |  |
| *Bembecia ichneumoniformis* | 15,323 | 78.7 | 3,701 | 76.2 | 766 | 85.8 | 1,285 | 83.7 | 1,441 | 82.1 | 537 | 94.2 | OU342551 | Boyes (2023) |
| *Synanthedon myopaeformis* | 15,176 | 78.7 | 3,697 | 76.3 | 762 | 85.4 | 1,289 | 84.0 | 1,440 | 82.3 | 401 | 95.5 | OX122944 | Unpublished |
| *Synanthedon andrenaeformis* | 16,650 | 80.1 | 3,701 | 76.4 | 762 | 85.2 | 1,295 | 82.6 | 1,448 | 82.4 | 1,746 | 96.1 | OW387807 | Unpublished |
| *Synanthedon formicaeformis* | 15,814 | 78.6 | 3,693 | 75.4 | 761 | 85.3 | 1,286 | 82.7 | 1,452 | 82.9 | 1,039 | 94.7 | OX243984 | Langdon and Fagan (2023) |
| *Synanthedon vespiformis* | 17,252 | 79.8 | 3,698 | 75.1 | 761 | 85.2 | 1,289 | 83.9 | 1,437 | 82.3 | 2,458 | 94.5 | OU906976 | Boyes and Lees (2022) |
| ***Synanthedon bicingulata*** | **16,255** | **79.7** | **3,697** | **76.7** | **759** | **85.0** | **1,288** | **85.1** | **1,451** | **82.4** | **759** | **94.1** | **PP622747** | **This study** |

^a^Termination codons for protein-coding genes (PCGs) were excluded from the total codon count.
