# Supplementary material for: Complete mitochondrial genome of the clearwing moth Synanthedon bicingulata (Lepidoptera: Sesiidae)
Source: Mitochondrial DNA B Resour. 2024 Nov 12;9(11):1528–32. doi: 10.1080/23802359.2024.2427095 (PMC11562021; doi:10.1080/23802359.2024.2427095)
Supplement: Figure S1_PCR.pdf [file TMDN_A_2427095_SM2352.pdf]

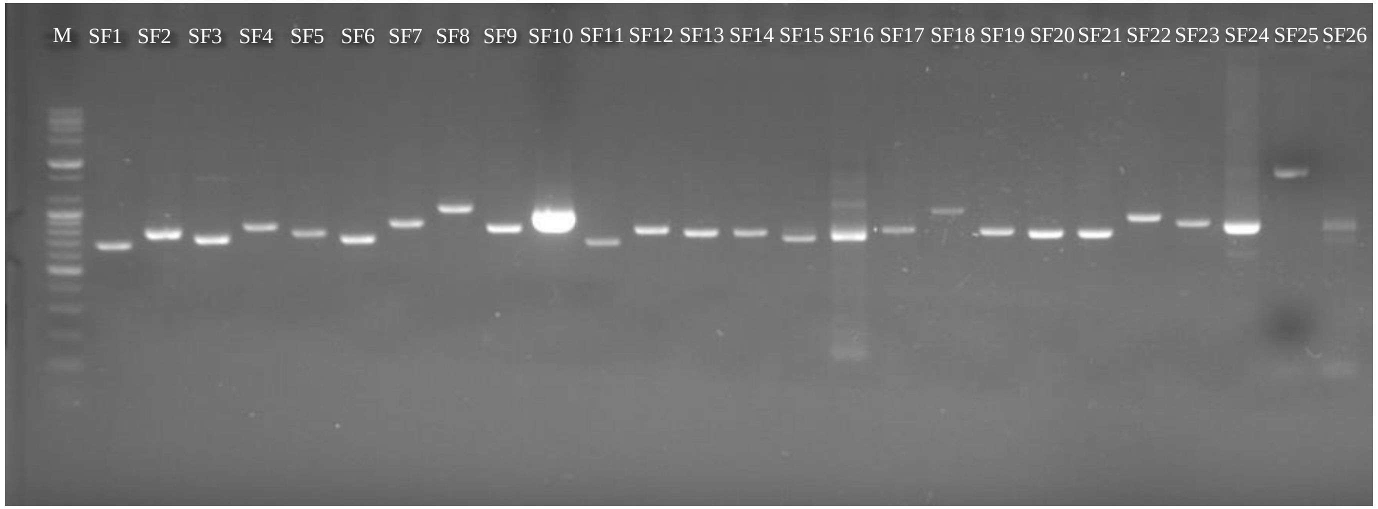

Figure S1. PCR products of 26 short overlapping fragments of *Synanthedon bicingulata* mitochondrial genome. The primer sequences are provided in Table S1. M indicates 100 bp plus marker.
