# Supplementary material for: Complete mitochondrial genome of the clearwing moth Synanthedon bicingulata (Lepidoptera: Sesiidae)
Source: Mitochondrial DNA B Resour. 2024 Nov 12;9(11):1528–32. doi: 10.1080/23802359.2024.2427095 (PMC11562021; doi:10.1080/23802359.2024.2427095)
Supplement: Figure S2_Linear arrangement_Revised.pdf [file TMDN_A_2427095_SM2350.pdf]

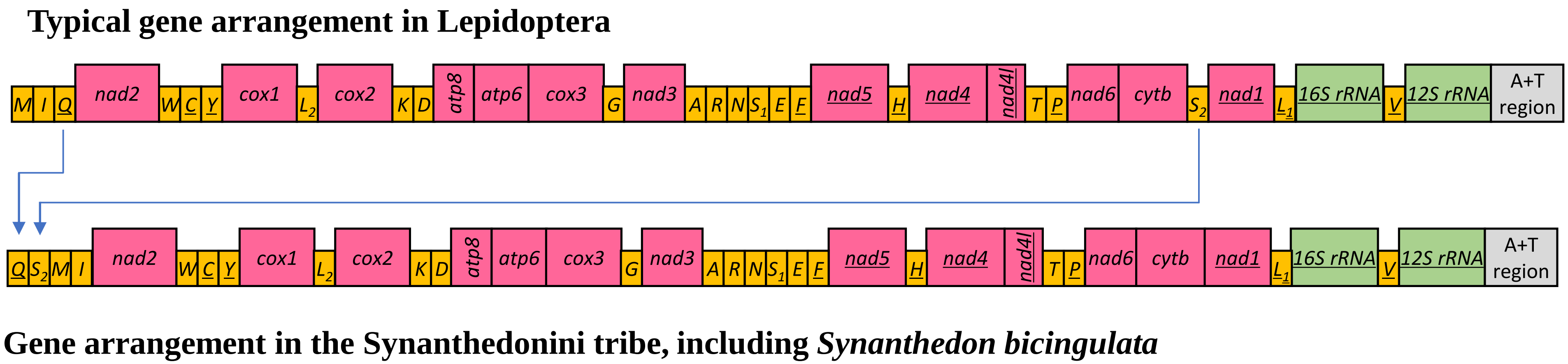

Figure S2. Schematic illustration of gene arrangement. Gene names that are not underlined indicate a clockwise transcriptional direction, excluding the A+T-rich region, whereas those that are underlined indicate a counter-clockwise transcriptional direction. Arrows indicate translocation position. tRNA abbreviations follow the IUPAC-IUB one-letter code. L_1, L_2, S_1, and S_2 denote $tRNA^{Leu}$(CUN), $tRNA^{Leu}$(UUR), $tRNA^{Ser}$(AGN), and $tRNA^{Ser}$(UCN), respectively. Gene sizes are not drawn to scale.
